# Complete Genome Sequence of Aeromonas hydrophila Strain Y-S01, Isolated from Diseased Dark Sleeper (Odontobutis potamophila) in China

**DOI:** 10.1128/mra.00933-22

**Published:** 2023-03-30

**Authors:** YanHua Zhao, ShiYong Zhang, HongYan Liu, Guoxing Liu

**Affiliations:** a Freshwater Fisheries Research Institute of Jiangsu Province, Nanjing, China; Montana State University

## Abstract

Aeromonas hydrophila is one of the most important pathogenic bacteria for aquaculture animals, such as fish and crustaceans. In this study, we isolated a pathogenic bacterial strain, named Y-SC01, from dark sleeper (Odontobutis potamophila) with rotten gills; the strain was identified as A. hydrophila by physiological and biochemical tests. Furthermore, we sequenced its genome and assembled a chromosome of 4.72 Mb with a GC content of 58.55%, and we report major findings based on the genomic analysis.

## ANNOUNCEMENT

Aeromonas hydrophila is a Gram-negative bacterium that is widely found in aquatic environments (1, 2) and is considered an opportunistic pathogen (3). It can cause infections such as septicemia, soft tissue infections, and gastroenteritis (4, 5).

We obtained rotten gill samples from diseased dark sleeper (Odontobutis potamophila) in Yangzhong, Jiangsu, China, disinfected and asepticized the body surface of the diseased fish, and separated the liver. Liver homogenates were cultivated on ordinary agar plates, which were incubated at 37°C in a constant-temperature incubator for 24 h. After purification, we isolated a bacterial strain named Y-SC01. PCR amplification was performed using 16S broad-spectrum primers (forward, 5′-AGTTTGATCMTGGCTCAG-3′; reverse, 5′-GGTTACCTTGTTACGACTT-3′). The PCR-amplified products were purified and sequenced by Sangon Biotech (Shanghai, China). The sequences obtained were aligned with a GenBank Basic Local Alignment Search Tool (BLAST) search. On the basis of the results of physiological and biochemical tests, strain Y-SC01 was eventually identified as Aeromonas hydrophila.

Dark sleeper fish were infected with strain Y-SC01 and showed the same symptoms as the diseased fish that were sampled. According to Koch's rule, strain Y-SC01 was the main cause of the rotted gills of the dark sleeper fish. To better understand the mechanisms underlying the diverse and highly useful biological activities of this strain, we sequenced and assembled its complete genome sequence.

Genomic DNA was isolated with a bacterial DNA kit (Omega Bio-Tek) according to the manufacturer’s instructions. The genome of strain Y-SC01 was sequenced using a combination of Pacific Biosciences (PacBio) RS and Illumina sequencing platforms. For the prokaryotic organism, we used an *ab initio* prediction method to obtain gene models for strain YSC01. Gene models were identified using GeneMark. All gene models were then searched with BLASTp against the nonredundant (NCBI), SwissProt (http://uniprot.org), KEGG (http://www.genome.jp/kegg), and COG (http://www.ncbi.nlm.nih.gov/COG) databases to perform functional annotation with the BLASTp module. In addition, tRNAs were identified using tRNAscan-SE v1.23 (http://lowelab.ucsc.edu/tRNAscan-SE), and rRNAs were determined using RNAmmer v1.2 (https://services.healthtech.dtu.dk/services/RNAmmer-1.2).

The Y-SC01 strain had a 4.72-Mb genome, with a GC content of 58.55%, consisting of one circular chromosome and one circular plasmid and containing 128 tRNAs (total length of 9,945 bp), 31 rRNAs (total length of 45,465 bp), and 4,364 genes (average gene length of 925 kb). The gene length was 4,716,245 bp, and the plasmid length was 7,435 bp. Among the 4,364 protein-coding genes, 1,752 Gene Ontology (GO) entries and 2,606 KEGG pathways were annotated successfully. The genome protein-coding density was 92.3%.

### Data availability.

The complete sequence of Y-SC01 is available in GenBank under accession number SRX15765507. BioProject accession no. PRJNA843574, SRA accession no. SRR19719023, and BioSample accession no. SAMN28748616.
